# Where is the Theoretical Basis for Understanding and Measuring the Environment for Physical Activity?

**DOI:** 10.4137/EHI.S1048

**Published:** 2008-12-02

**Authors:** N.M. Nelson, A. Wright, R.G. Lowry, N. Mutrie

**Affiliations:** 1 Department of Sport, Culture and the Arts, University of Strathclyde, Glasgow, UK; 2 Sport, Exercise and Health Sciences, University of Chichester, West Sussex, UK

**Keywords:** environment, physical activity, theory, measurement

## Abstract

Researchers are beginning to explore environmental correlates to further the field of physical activity research. Before interventions and experimental investigations can be undertaken, it is necessary to identify specific environmental features that are consistent correlates of physical activity. There has been a plethora of research measuring such cross-sectional associations since this field came to the fore in 2003. This paper posits that it is time for researchers to evaluate the state of knowledge, and suggests that future developments in this field focus on the theoretical bases for (i) measurement of the environment and (ii) understanding the links between perceptions of the environment and behaviour through psychological theories of cognition. Key theories considered include social ecology and the theory of planned behaviour. It is suggested that with a continued absence of a common conceptual framework, vocabulary and measurement tools the majority of studies may remain at a correlates stage. In highlighting issues with current methodologies, this commentary encourages more grounded theoretical approaches to the study of the environment and physical activity.

## Introduction

The accumulation of at least thirty minutes of moderate intensity activity, on at least five days of the week, is related to numerous mental and physical health benefits for adults (Department of Health [DOH] 2004; Haskell et al. 2007). In spite of these advantages, the majority of the population in developed countries do not achieve this standard (DOH 2004; Scottish Executive 2005; U.S. Department of Health and Human Services 1996). Personal, environmental, social, economic and political factors have all been shown to exert an effect on the activity levels of individuals and communities (Dishman and Sallis, 1994; Sallis and Owen, 1999) but each explain only a small proportion of the variance in activity levels (Sallis and Owen, 1999). Understanding the individual in context is crucial, and this includes social, cultural, political and physical environments.

In the field of physical activity research, studies are emerging that suggest a definite role for the physical environment in some form (Booth et al. 2001; Giles-Corti et al. 2005; Humpel et al. 2002; Owen et al. 2004; Saelens et al. 2003; Sallis et al. 1997). Recognition of environmental influences has also occurred at UK government level; specifically, both the Chief Medical Officer’s report in England (Department of Health 2004) and the Foresight Report (Government Office for Science 2007) have acknowledged the mediating role of the environment in terms of physical activity behaviour. The National Institute for Health and Clinical Excellence (NICE) has recently issued guidelines considering the promotion and creation of physical environments that support increased levels of physical activity (NICE 2008), and there is a need for well-designed population and behaviour specific research that seeks to clarify the environment-physical activity relationship in order to guide the development of interventions.

There are a number of potential advantages for an improved understanding of the role of environment in the decision to be (in) active. The environmental attributes of an area such as its structure, design and physical features will influence the lives of all who live in the area, and this includes providing cues and opportunities for physical activity (Giles-Corti and Donovan, 2002; Jackson and Kochtitzky, 2003). Any social, economic or physical environmental facilitators or inhibitors of physical activity will therefore impact at the community level and act across populations (Brownson et al. 2008). Modifications to the environment have the potential for much longer-lasting effects than individual level interventions because changes are assimilated into structures, systems, policies and socio-cultural norms (Broderson et al. 2005; De Bourdeaudhuij et al. 2003; Owen et al. 2000; Shenassa, 2001).

Research into environmental impacts on physical activity has increased exponentially since this field came to the fore in 2003 (Dannenberg et al. 2003), although the majority of currently available literature is descriptive and from cross sectional studies, with researchers seeking to in some way identify and quantify possible relationships. In a recent critique of the synthesis of evidence, Gebel, Baumann and Petticrew (2007) suggest that the quality of reviews varies and significant methodological variation ensures that comparison and extraction of overall conclusions remains challenging (Gebel et al. 2007). This commentary posits that it is time for researchers to evaluate the state of knowledge, and suggests that future developments in this field focus on the theoretical bases for (i) measurement of the environment and (ii) understanding the links between perceptions of the environment and behaviour through psychological theories of cognition.

## The Theoretical Basis for Measurement of the Environment

The concept that environmental factors can hinder or facilitate desired behaviour change is a valuable, albeit recent, adjunct to the individual-behavioural theories in early health promotion research (Sallis et al. 1998; Humpel et al. 2002; Sallis and Owen, 1999). Since the physical environment became the focus of physical activity research (Dannenberg et al. 2003), social ecological theory has been the most commonly adopted theoretical framework for research (Humpel et al. 2002). Social ecology involves the social, cultural, and institutional contexts of people-environment relations (Stokols, 1992), referring to elements largely outside an individual’s control but modifiable by society (Sallis et al. 1997). It has been suggested that intrapersonal, physical environmental, social and cultural variables interact and have both direct and indirect effects on health behaviours; such effects may differ by population sub group, geographical setting or other contextual factors (Sallis et al. 1997). Although it intuitively appeals as a real-world model, the multilevel, all-encompassing nature belies empirical testing and few researchers have proposed, developed or tested social ecological models of physical activity behaviour. More frequently, researchers have relied on social ecology to support a new emphasis on research into external, environmental variables (Humpel et al. 2002). However, ecological models lack specificity about which characteristics of the environment might influence behaviour (Humpel et al. 2002), forcing researchers to look elsewhere for conceptualisations of the environment and how to measure it.

We suggest that an examination of three influential articles in this field sheds light on the development of commonly used measures and methods. In 2002, Humpel and colleagues examined nineteen quantitative studies of environmental influences on physical activity, all conducted by researchers from the physical activity field. Measures were mostly self-report questionnaires on perceptions of the environment which were poorly developed or reported, and selected based on pragmatic insights of the researchers rather than previous research or theory. Nonetheless, five ‘groupings’ of environmental variables emerged: accessibility of facilities, opportunities for activity, weather, safety and aesthetics. The authors noted that social ecological theory was commonly cited, and thus recommended behaviour- and context-specific measurement strategies in future research.

The second key article, by Pikora and colleagues (2002) presents the development of a framework of potential environmental influences on walking and cycling based on policy literature, interviews with experts and a Delphi study—a systematic, interactive forecasting method which relies on a panel of independent experts. The authors report some disagreement about relative importance of the environmental influences between the experts who represented urban planning/local government, transport, public health and physical activity advocacy groups. The final framework included four features (function, safety, aesthetics and destinations), the hypothesised factors that contribute to these features and their relative importance (Pikora et al. 2002). This framework added detail and expanded the scope of potential environmental variables in the physical activity field, while retaining the emphasis on behaviour specificity emphasised by ecological theory.

Finally, the third article by Saelens and colleagues (2003) emphasised the importance of research from the transportation and planning literature and its associated methodology and terminology (e.g. land use, connectivity, walkability) to the physical activity field. Incorporating this evidence, the Neighbourhood Environment Walkability Scale (NEWS) was developed to measure perceptions of the physical environment (Saelens et al. 2003) and this tool has since been applied worldwide (Cerin et al. 2007; Cerin et al. 2008; Fitzsimons et al. 2008; Woods et al. 2008). The concept of ‘spatial multicollinearity’ of environmental features was introduced, whereby environmental characteristics such as land use patterns, transportation systems and neighbourhood design coexist and are inter-related. In addition, the need to address potential confounders such as socio-demographics was discussed, again emphasising characteristics of social ecological theory.

These three articles clearly illustrated how physical activity researchers have moved from relying on pragmatic insights towards consultation with experts and acceptance of methodology and terminology from the transportation and planning fields. However, evidence has continued to emerge under the separate strands of urban planning/travel behaviour and public health/physical activity, with their respective theoretical and methodological emphases and consequently sometimes contradictory results. Comprehensive reviews have been conducted by professionals from both strands (Badland and Schofield, 2005; Frank et al. 2003; Humpel et al. 2002; McCormack et al. 2007; Owen et al. 2004; Saelens et al. 2003; Sallis et al. 2004) and the reader is referred to these for a detailed analysis of findings to date. Our summary of these findings suggests that research from the urban planning and transportation fields has focused primarily on destinations-oriented walking and non-motorised travel, using objective measures of the environment (for example geographical information systems), to measure neighbourhood type (e.g. traditional/transit-oriented/walkable), land use mix, grid-like street networks and functional infrastructure. In contrast, research from the physical activity and public health arena has primarily focused on recreational walking or physical activity, often including perceptual characteristics in place of, or alongside, objective measures of the environment. Consequently, results commonly focus on subjective variables such as safety and aesthetics. Drawing on ecological theory, many researchers have included personal, social and/or socioeconomic variables in their analyses, but despite early recommendations to examine the relative influence of these as against the environment (Giles-Corti and Donovan, 2002) this is only recently becoming more commonplace.

It appears that researchers from both strands have become conversant in the terminology of the other and, in some cases, have adopted measurement tools and methods. However, the lack of theoretical grounding pervades both strands, resulting in a plethora of exploratory research, that assumes only direct effects on physical activity, with no interaction between environmental variables. Few studies in either strand have examined the relative importance of environmental characteristics in multivariate models, or their potential confounding, mediating and moderating interactions (Li et al. 2005). As this field develops, it is important to recognise the limitations of analysing the effect of individual features of the environment on physical activity behaviour. These features do not exist in isolation and any effects measured or analysed in this manner are likely to be unrealistic. The recognition of multicollinearity between environmental variables (Carver et al. 2005; Nelson and Woods, 2008) will provide researchers with measurement challenges to overcome. It is important that these challenges are addressed and the complexity of the environment is embraced; recommendations and interventions based on poorly designed research risk the undermining of genuine effects, and the loss of confidence in the importance of the environment as a potential solution to physical inactivity. Future research can address these issues with improved study design that includes variable neighbourhood features and addresses potential confounders such as socio-demographics, as was recommended by Saelens and colleagues in 2003; however, it seems that this issue has been overlooked in recent research. In the absence of an integrated theoretical framework at the design level, researchers should consider sophisticated data analyses that allow for multilevel modelling and interactions between environmental features, such as structural equation modelling (SEM). Recent research has indicated that a combination of factor analysis and SEM can be used to describe a theoretically meaningful framework accounting for interrelationships between environmental variables while explaining a large proportion of variance in active commuting behaviour (Nelson and Woods, 2008). The use of such data driven statistical models in such software may allow existing theories from alternative fields to be tested and assist in the development of new theories based on the large masses of data already gathered in this field.

## The Theoretical Basis for Linking Perceptions of the Environment and Behaviour

Despite the body of evidence suggesting a link between the environment and physical activity, there is a conspicuous absence of a theoretical framework through which to understand how environmental correlates influence an individual’s behavioural intention or change. This is evident in the common unanswered questions posed by researchers, such as: Is building or creating an activity-enhancing environment sufficient for individuals to become active? (Giles-Corti et al. 2007); Do individuals self-select into activity-enhancing environments? (Handy et al. 2006); Does a hierarchy of environmental correlates exist that would indicate the decision-making processes of the individual who wants to become more physically active? (Alfonzo, 2005); How or why do potential changes in perceived environment result from physical activity changes? (Humpel et al. 2004). Consideration also needs to be given to developmental differences in explanations of an individual’s interaction with their environment; explanations of adult choices are perhaps not appropriate for children who have relatively little control over choices in their environment. Psychologists are interested in studying interactions between humans and their environment. Thus, by examining psychological theories, we may begin to establish a framework in which the current emergence of environmental research should be referenced to answer wider issues of sustaining healthy behaviours.

The theory of planned behaviour (TBP, Ajzen 1985, 1988; Ajzen and Madden, 1986), a progression from the earlier theory of reasoned action (Fishbein, 1967) is worth consideration. It seeks to provide a framework to understand and, more crucially, predict the associations between attitudes and behaviour. Although first developed to understand and predict disease related behaviours such as alcohol consumption, the TPB has been used extensively to predict physical activity participation (Biddle and Mutrie, 2008; Ogden, 2007). The theory posits that an individual’s behaviour is determined primarily by his or her intention to engage in the behaviour. This behavioural intention is itself predicted by attitudes regarding the behaviour, subjective norms from the social environment and the level of perceived behavioural control the individual believes he or she can exert. The TPB may be used to elucidate the links between environmental cognitions and physical activity and provide valuable insight into *how* an individual’s decision of whether or not to be active is influenced by a supportive physical environment.

As an example, Rhodes et al. (2006) investigated the mediating and moderating variables of perceptions of the physical environment and the TPB. They concluded that perceived environmental factors are antecedents to physical activity motivation but do not have an effect on behaviour independent of individual motivation. However, they do concede that aesthetics and land use mix are likely to influence walking attitudes, and potentially walking behaviour, by means of intention (Rhodes et al. 2006). Further, along with intention, perceived proximity to retail destinations was the only physical or social environmental construct to independently predict walking in a fully adjusted model of activity in Canadian adults (Rhodes et al. 2007). In a practical sense, these results imply that more and better quality green space and streetscapes and a convenient mix of land uses may help in the transition of intentions into behaviour, and that individuals who live closer to retail destinations may end up walking more than originally intended; however, such environmental issues are not sufficient to directly promote physical activity behaviour. The majority of researchers have failed to acknowledge that the impact of a supportive environment is primarily determined by individual cognitions. If a person cognitively assesses any one of the components of the TPB as negative then this would help to predict why the planned behaviour does not translate into positive action, even if the person’s environment is conducive towards physical activity. Perhaps an individual is not in a financial position to move; perhaps they don’t have the support of significant others; or perhaps they fail to value the role of the neighbourhood in activity promotion (choosing to exercise at a gym instead). Using the structure of the theory of planned behaviour it might be feasible to plot the underlying norms and attitudes surrounding other behaviours such as active commuting. This is of course the tip of a very large theoretical iceberg; the TPB is only one of many explanations of behaviour, motivation and attitudes available to researchers. Other plausible theories might include the transtheoretical model (Marcus and Simpkin, 1994) or self determination theory (Deci and Ryan, 1985); however, there is no consensus as to which theory best predicts behaviour (Biddle and Mutrie, 2008).

## Conclusions

Due to methodological flaws largely resulting from a lack of theoretical grounding, it is currently difficult to say which characteristics of the environment have the strongest associations with physical activity or how strong these associations are. There remains a need to identify the mechanisms behind observed relationships and the relative importance of the environment compared to other factors of influence. However, in the absence of a common conceptual framework and a more standardised vocabulary and measurement tools, the majority of studies will remain at a correlates stage. To move this area of enquiry forward, researchers must first pause and re-consider grounding their investigations in theoretical foundations.
